# Development of a novel prognostic assessment model for hepatitis B virus-related acute-on-chronic liver failure based on reexamination results

**DOI:** 10.1097/MD.0000000000033252

**Published:** 2023-03-17

**Authors:** Dakai Gan, Yuyu Zeng, Kaige Zhang, Yang He, Jiao Wan, Xiaoqing Zhang, Zhen Zhang, Longchuan Zhu, Tao Long, Nengwen Xie, Bo Zou, Xuezhen Zhang, Yunfeng Xiong, Guoliang Feng, Daya Luo, Molong Xiong

**Affiliations:** a Infectious Diseases Hospital Affiliated to Nanchang University, Nanchang City, China; b Third Clinical Medical College Affiliated to Nanchang University, Nanchang City, China; c School of Medicine, Nanchang University, Nanchang City, China.

**Keywords:** acute-on-chronic liver failure, hepatitis B, new model, prognosis

## Abstract

Acute-on-chronic liver failure (ACLF) is a common clinical emergency and critical illness with rapid progression and poor prognosis. This study aims to establish a more efficient system for the prognostic assessment of hepatitis B virus-related acute-on-chronic liver failure (HBV-ACLF), which will provide a guiding scheme for subsequent treatment and improve the survival rate of patients. Data on 623 patients with HBV-ACLF were recorded. Univariate and multivariate analyses were performed to determine the discriminative abilities of the novel prognostic assessment model in predicting 90-day mortality. The area under the receiver operating characteristic curve was used to evaluate the accuracy of the models. Patients were divided into high- and low-scoring groups based on the best critical values, and survival rates were analyzed using Kaplan–Meier survival analysis and compared by applying log-rank tests. The area under the curve of the new scoring system established using the results of the first reexamination, the results of the first examination, the mean daily change in these results (MDCR) and the results of other first examinations were 0.911 (95% confidence interval [CI]: 0.889, 0.933), 0.893 (95% CI: 0.868, 0.917), and 0.895 (95% CI: 0.871, 0.919), respectively. The final prognostic scoring system established using the results of the first reexamination was chosen as a novel prognostic assessment model, and patients with lower scores (first reexamination results [FRER] score ≤ 3.65) had longer survival times (*P* < .001). The prognostic scoring system established using the FRER combined with other examination results can better assess the prognosis of HBV-ACLF at 90 days.

## 1. Introduction

Acute-on-chronic liver failure (ACLF) is distinct from chronic liver failure or decompensated cirrhosis based on the presence of a precipitating factor: rapid deterioration leading to liver and/or extrahepatic multiorgan failure with high short-term (28 days and 90 days) mortality. Based on the underlying liver disease, the World Gastroenterology Organization further classified ACLF into 3 categories: type A ACLF (patients with noncirrhotic chronic liver disease), type B ACLF (patients with compensated cirrhosis) and type C ACLF (patients with decompensated cirrhosis).^[1]^ The diagnosis of ACLF is related to the short-term prognosis. Compared with ACLF type A and type B patients, ACLF type C patients have the worst prognosis. Consortium Acute-on-Chronic Liver Failure in Cirrhosis research data show that the overall 28-day mortality rate of ACLF is 33% while the 28-day mortality rates of ACLF types A, B, and C are 22%, 32%, and 73%, respectively.^[2]^ As seen from the above, ACLF is a common clinical acute and critical illness that exhibits rapid progression and poor prognosis. Therefore, assessing the severity of the condition of patients with liver failure at an early stage, predicting the return of the condition, and adjusting the treatment plan in time is of great significance for improving patient prognosis.^[3]^

In recent years, efforts of scholars worldwide have led to the development of various models for assessing the prognosis of liver failure to guide the development of clinical treatment strategies. According to the study data, the overall prognostic indicators can be divided into single-factor clinical prognostic indicators and multifactor clinical prognostic indicators. Single-factor clinical prognostic indicators include age, etiology, transaminases, serum ferritin, prothrombin time (PT), international normalized ratio (INR), prothrombin activity (PTA), and methemoglobin.^[4–6]^ However, due to the organic wholeness of the human body and the complexity of the disease itself, various factors intermingled with each other and single factors often do not provide a good prognosis for ACLF. Therefore, multifactorial prognostic indicators are more frequently used in clinical practice, including the model of end-stage liver disease (MELD) and its derived assessment models (MELD-Na, MESO, iMELD, uMELD, MELD-CD, UKMELD, D-MELD, etc) and the sequential organ failure assessment model and its derived assessment models (CLIF-sequential organ failure assessment model, CLIF C, CLIF-C ADs, CLIF-C OFs), CTP and mCTP scores.^[7–10]^ Nevertheless, multifactorial prognostic indicators have certain limitations. For example, all of the above scoring models are based on the initial examination results, which do not fully reflect the progression of the disease. Various factors lead to the limited accuracy of these scores in predicting the prognosis of ACLF, and a widely accepted scoring system is not available to guide physicians in clinical practice. Therefore, the establishment of a more efficient, accurate, and convenient assessment system for the prognostic evaluation of hepatitis B virus (HBV)-ACLF would provide a guiding scheme for subsequent treatment, improve patient survival, and maximize the quality of patient survival.

This study evaluated the impact of progress or changes in corresponding indicators on the prognosis of the disease, including the first examination results (FER), the first reexamination results (FRER), general conditions, and complications that occurred during the course of the disease, screened out independent risk factors, and analyzed the selected independent risk factors separately. A new prognostic evaluation model of HBV-ACLF was obtained.

## 2. Patients and Methods

### 2.1. Research object

**Figure 1.**
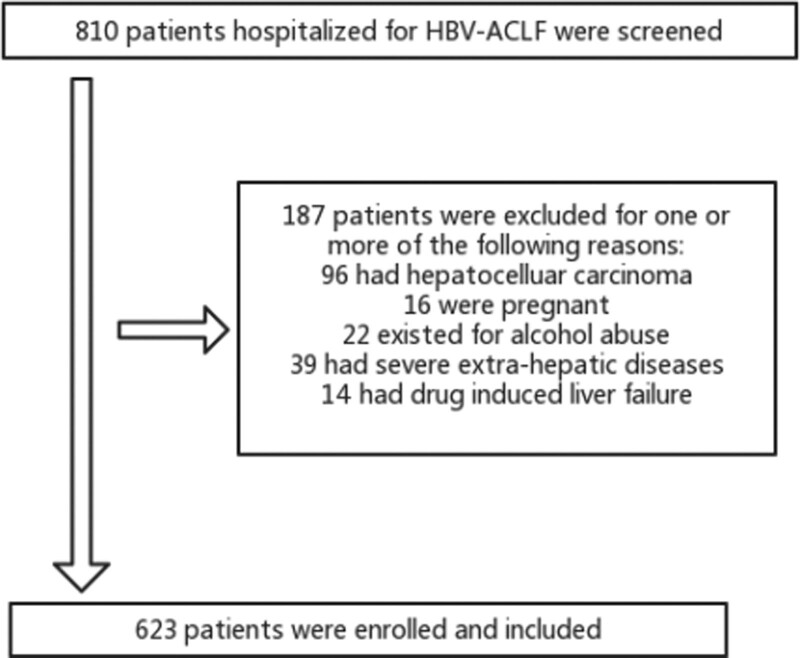
Outline of the screening and case selection protocol.

Clinical data on 810 cases of HBV-ACLF treated at our hospital from January 2010 to December 2020 were retrospectively analyzed, 187 patients did not meet the inclusion and exclusion criteria (Fig. 1). The FER, FRER, general conditions, and complications that occurred during the course of the disease were recorded. ACLF was diagnosed according to the Asian Pacific Association for the Study of the Liver (APASL):^[11]^ jaundice (serum total bilirubin [TBil] ≥ 85 μmol/L), coagulopathy (INR ≥ 1:5 or PTA ≤ 40%), and any degree of encephalopathy and/or clinical ascites within 4 weeks for ongoing chronic liver diseases. Inclusion criteria: All inpatients with HBV-related ACLF admitted to our hospital from January 2010 to December 2020 who were confirmed to be positive for HBsAg or HBV DNA through hematology and immunology tests were selected. Exclusion criteria: co-infection with other types of hepatitis viruses; combined hepatocellular carcinoma; obstructive jaundice and hemolytic jaundice due to common bile duct stones; simple alcoholic fatty liver or nonalcoholic fatty liver; autoimmune-related liver disease; drug-related liver injury; preexisting serious heart, brain, lung, kidney and other vital organ diseases; systemic chronic or metabolic diseases such as hyperthyroidism, tuberculosis, and tumors; missing clinical data or all test data. The observation period lasted for 90 days. The start of the survival period for all cases was the day of diagnosis, and the end point of the survival period for patients lost to follow-up was the date of the last follow-up visit.

### 2.2. Patient management

On the 3rd to 7th days after admission, the patient hematological indicators were reviewed for the first time. During this period, all patients received standard medical treatment, including bed rest, intravenous antibiotics, liver protective treatment, and energy supplements, to actively prevent and treat complications. Antiviral therapies, including lamivudine, telbivudine, adefovir dipivoxil, or entecavir, were administered individually according to the virus replication levels and patients’ conditions. Subsequently, the corresponding artificial liver model was selected for treatment according to the specific circumstances.

### 2.3. Statistical analysis

Statistical analyses were performed using SPSS software (version 26.0; IBM Corporation, Somers, NY). The measurements are presented as quartiles (*P*_*25*_, *P*_*75*_), means ± (*SD*) or numbers (%), unless otherwise noted. The independent prognostic factors were identified by multivariate Cox regression analysis, and a new prognostic scoring system was established based on the Cox proportional hazard regression. The area under the receiver operating characteristic (ROC) curve was used for model discrimination and calibration. A comparison of the cumulative survival rates was conducted with the Kaplan–Meier method. Differences were considered statistically significant at *P* < .05.

### 2.4. Ethical statements

The study used medical records obtained from previous clinical consultations, met all of the following conditions, and was exempted from informed consent by the Ethics Committee of Nanchang Number 9 Hospital. The ethical approval number was [2022] Lunjian Audition No. (03).

1. The purpose of the study is clear;
2. The risk to the subject of the study is not greater than the minimum risk;
3. The exemption of informed consent will not adversely affect the rights and health of the subject;
4. The privacy and personally identifiable information of the subjects be protected.

## 3. Results

### 3.1. Characteristics and outcomes of HBV-ACLF patients

Our study included 623 patients. Table 1 reveals the baseline characteristics of the HBV-ACLF patients. During a 90-day follow-up, 305 patients (48.96%) were deceased or had received a liver transplant.

**Table 1:** Clinical characteristics of the enrolled patients.

Parameters	All patients (n = 623)
Age (yr)	45.0 (37.0–54.0)
Male, n (%)	547 (87.8%)
ALT (IU/L)	425.0 (142.0–941.0)
AST (IU/L)	297.9 (136.0–700.0)
Alb (g/L)	31.3 (27.6–34.4)
Talb (g/L)	61.9 (56.5–68.9)
TBIL (μmmol/L)	317.9 (220.8–419.7)
INR	2.3 (1.8–3.1)
PT (s)	26.2 (21.2–33.0)
PTA (%)	34.0 (25.0–45.0)
HB (g/L)	126.0 (109.0–139.0)
WBC (10^9^/L)	6.3 (4.6–8.5)
PLT (10^9^/L)	98.0 (64.8–138.3)
BUN (mmol/L)	3.9 (2.9–5.4)
Cre (μmmol/L)	65.0 (55.0–80.0)
Na (mmol/L)	137.2 (134.2–139.6)
AFP (ng/mL)	43.2 (9.6–147.3)
Ascites, n (%)	487 (78.2%)
Infectious, n (%)	428 (68.7%)
HE, n (%)	202 (32.4%)
UGIB, n (%)	66 (10.6%)
ALS, n (%)	465 (74.6%)

Data are presented as n (%) or median (interquartile range).

AFP = alpha-fetoprotein, Alb = albumin, ALS = artificial liver support system, ALT = alanine aminotransferase, AST = aspartate aminotransferase, BUN = blood urea nitrogen, Cre = creatine, HB = haemoglobi, HE = hepatic encephalopathy, INR = international normalized ratio, PLT = platelet, PT = prothrombin, PTA = prothrombin activity, TBil = total bilirubin, TP = total protein, UGBI = upper gastrointestinal hemorrhage, WBC = white blood cell.

### 3.2. Independent prognostic factors and development of 3 new predictive models

**Figure 2.**
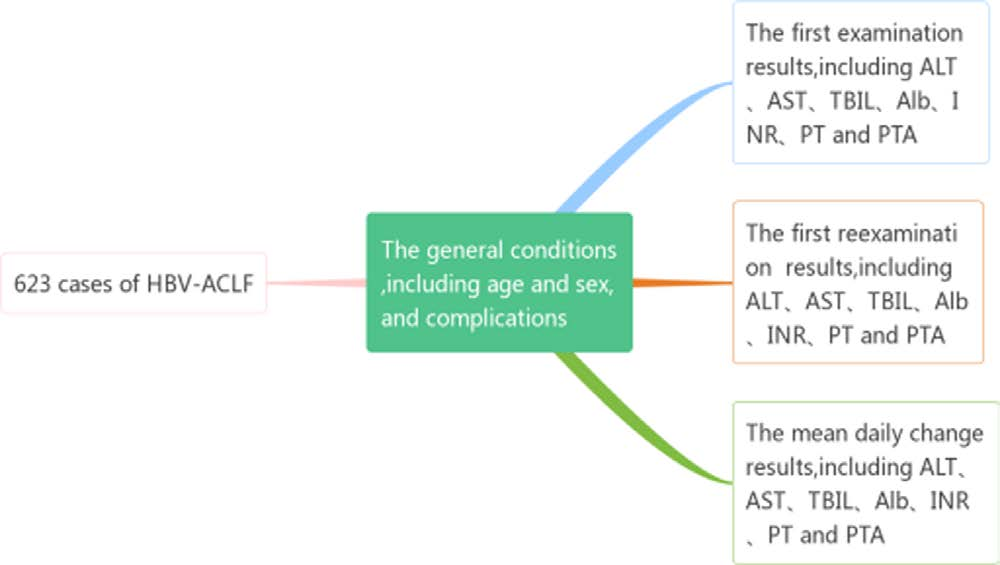
Indicators to be included in the 3 new prognostic scoring systems.

The FRER, FER, mean daily change in these results (MDCR), general conditions, including age and sex, and complications, including ascites, infection, hepatic encephalopathy (HE), and upper gastrointestinal bleeding, were included as model parameters (Fig. 2). After performing the univariate Cox regression, clinically significant parameters were verified by multivariate analysis (Tables 2, 3 and 4). Then, 3 new prognostic models for HBV-ACLF patients were established using the following mathematical formulas (FER score, FRER score, and MDCR score):

**Table 2:** Univariate and multivariate Cox regression analyses of 90-day mortality based on the first examination results.

	*P*	*B*	Univariate analyses HR (95% CI)	*P*	*B*	Multivariate analyses HR (95% CI)
ALT	.354	0.000	1.000 (1.000, 1.000)			
AST	.241	0.000	1.000 (1.000, 1.000)			
TBIL	<.001	0.002	1.002 (1.001, 1.003)	.001	0.006	1.001 (1.000, 1.002)
ALB	.356	0.000	1.000 (1.000, 1.000)			
INR	<.001	0.304	1.355 (1.289, 1.424)	.875	0.018	1.018 (0.817, 1.269)
PT	<.001	0.038	1.039 (1.032, 1.045)	.344	0.014	1.014 (0.985, 1.045)
PTA	<.001	−0.048	0.953 (0.944, 0.963)	.024	−0.019	0.981 (0.965, 0.998)
Gender	.895	0.023	1.023 (0.726, 1.443)			
Age	<.001	0.027	1.027 (1.018, 1.036)	<.001	0.026	1.026 (1.016, 1.036)
Talb	.099	−0.011	0.989 (0.977, 1.002)			
Bun	<.001	0.112	1.119 (1.092, 1.146)	.004	0.062	1.064 (1.020, 1.110)
Cre	<.001	0.007	1.007 (1.005, 1.009)	.831	0.000	1.000 (0.997, 1.003)
Na	<.001	−0.014	0.986 (0.980, 0.993)	.190	−0.009	0.991 (0.979, 1.004)
Wbc	<.001	0.116	1.122 (1.096, 1.150)	<.001	0.061	1.063 (1.033, 1.094)
Hb	<.001	−0.001	0.99 (0.985, 0.994)	.001	0.010	1.010 (1.004, 1.015)
Plt	.161	−0.002	0.998 (0.996, 1.001)			
AFP	<.001	−0.003	0.997 (0.996, 0.998)	.008	−0.001	0.999 (0.998, 1.000)
ascites	<.001	1.541	4.669 (3.023, 7.209)	.627	−0.180	0.835 (0.403, 1.729)
Infection	<.001	1.792	6.003 (4.090, 8.812)	.003	0.996	2.707 (1.414, 5.192)
HE	<.001	1.735	5.667 (4.489, 7.155)	<.001	1.127	3.087 (2.384, 3.996)
Ugib	<.001	0.972	2.642 (1.970, 3.543)	.529	0.100	1.105 (0.809, 1.510)

AFP = alpha-fetoprotein, Alb = albumin, ALT = alanine aminotransferase, AST = aspartate aminotransferase, BUN = blood urea nitrogen, CI = confidence interval, Cre = creatine, HB = hemoglobin, HE = hepatic encephalopathy, HR = hazard ratio, INR = international normalized ratio, PLT = platelet, PT = prothrombin, PTA = prothrombin activity, TBil = total bilirubin, TP = total protein, UGBI = upper gastrointestinal hemorrhage, WBC = white blood cell. After univariate Cox regression, clinically significant parameters were verified by multivariate analysis.

**Table 3:** Univariate and multivariate Cox regression analyses of 90-day mortality based on the first reexamination results.

	*P*	*B*	Univariate analyses HR (95% CI)	*P*	*B*	Multivariate analyses HR (95% CI)
ALT	.046	0.000	1.000 (1.000, 1.000)	.936	0.000	1.000 (1.000, 1.000)
AST	<.001	0.001	1.001 (1.001, 1.001)	.037	0.001	1.001 (1.000, 1.001)
TBIL	<.001	0.003	1.003 (1.002, 1.004)	<.001	0.002	1.002 (1.001, 1.003)
ALB	.302	0.000	1.000 (1.000, 1.000)			
INR	<.001	0.057	1.058 (1.041, 1.075)	.214	0.026	1.026 (0.985, 1.069)
PT	<.001	0.050	1.051 (1.045, 1.057)	.011	0.019	1.019 (1.004, 1.034)
PTA	<.001	−0.065	0.937 (0.927, 0.946)	.001	−0.029	0.971 (0.956, 0.987)
Gender	.895	0.023	1.023 (0.726, 1.443)			
Age	<.001	0.027	1.027 (1.018, 1.036)	<.001	0.026	1.026 (1.016, 1.037)
Talb	.099	−0.011	0.989 (0.977, 1.002)			
Bun	<.001	0.112	1.119 (1.092, 1.146)	<.001	0.078	1.081 (1.036, 1.129)
Cre	<.001	0.007	1.007 (1.005, 1.009)	.548	−0.001	0.999 (0.980, 1.007)
Na	<.001	−0.014	0.986 (0.980, 0.993)	.349	−0.007	0.994 (0.980, 1.007)
Wbc	<.001	0.116	1.122 (1.096, 1.150)	<.001	0.059	1.061 (1.032, 1.091)
Hb	<.001	−0.001	0.99 (0.985, 0.994)	.201	0.004	1.004 (0.998, 1.009)
Plt	.161	−0.002	0.998 (0.996, 1.001)			
AFP	<.001	−0.003	0.997 (0.996, 0.998)	.207	-0.001	0.999 (0.998, 1.000)
ascites	<.001	1.541	4.669 (3.023, 7.209)	.172	-0.505	0.603 (0.292, 1.246)
Infection	<.001	1.792	6.003 (4.090, 8.812)	.001	1.123	3.075 (1.594, 5.930)
HE	<.001	1.735	5.667 (4.489, 7.155)	<.001	0.839	2.314 (1.759, 3.044)
Ugib	<.001	0.972	2.642 (1.970, 3.543)	.754	0.05	1.051 (0.770, 1.435)

AFP = alpha-fetoprotein, Alb = albumin, ALT = alanine aminotransferase, AST = aspartate aminotransferase, BUN = blood urea nitrogen, CI = confidence interval, Cre = creatine, HB = hemoglobin, HE = hepatic encephalopathy, HR = hazard ratio, INR = international normalized ratio, PLT = platelet, PT = prothrombin, PTA = prothrombin activity, TBil = total bilirubin, TP = total protein, UGBI = upper gastrointestinal hemorrhage, WBC = white blood cell. After univariate Cox regression, clinically significant parameters were verified by multivariate analysis.

**Table 4:** Univariate and multivariate Cox regression analyses of 90-day mortality based on the mean daily change in these results.

	*P*	*B*	Univariate analyses HR (95% CI)	*P*	*B*	Multivariate analyses HR (95% CI)
ALT	.870	0.000	1.000 (0.999, 1.001)			
AST	.543	0.000	1.000 (1.000, 1.001)			
TBIL	<.001	0.011	1.011 (1.006, 1.016)	.017	0.007	1.007 (1.001, 1.012)
ALB	.297	0.008	1.008 (0.993, 1.024)			
INR	<.001	0.208	1.231 (1.122, 1.352)	.442	0.077	1.080 (0.888, 1.314)
PT	<.001	0.059	1.061 (1.044, 1.078)	<.001	0.125	1.133 (0.091, 1.176)
PTA	.023	−0.055	0.947 (0.903, 0.992)	<.001	−0.150	0.861 (0.806, 0.919)
Gender	.895	0.023	1.023 (0.726, 1.443)			
Age	<.001	0.027	1.027 (1.018, 1.036)	<.001	0.022	1.023 (1.012, 1.033)
Talb	.099	−0.011	0.989 (0.977, 1.002)			
Bun	<.001	0.112	1.119 (1.092, 1.146)	.001	0.073	1.076 (1.031, 1.121)
Cre	<.001	0.007	1.007 (1.005, 1.009)	.249	−0.002	0.998 (0.995, 1.001)
Na	<.001	−0.014	0.986 (0.980, 0.993)	.274	−0.007	0.993 (0.982, 1.005)
Wbc	<.001	0.116	1.122 (1.096, 1.150)	<.001	0.075	1.078 (1.048, 1.108)
Hb	<.001	−0.001	0.99 (0.985, 0.994)	.005	0.054	1.005 (1.000, 1.011)
Plt	.161	−0.002	0.998 (0.996, 1.001)			
AFP	<.001	−0.003	0.997 (0.996, 0.998)	.018	−0.001	0.999 (0.998, 1.000)
ascites	<.001	1.541	4.669 (3.023, 7.209)	.328	−0.352	0.703 (0.347, 1.424)
Infection	<.001	1.792	6.003 (4.090, 8.812)	.001	1.096	2.992 (1.594, 5.616)
HE	<.001	1.735	5.667 (4.489, 7.155)	<.001	1.194	3.301 (2.548, 4.277)
Ugib	<.001	0.972	2.642 (1.970, 3.543)	.719	−0.059	0.943 (0.685, 1.299)

AFP = alpha-fetoprotein, Alb = albumin, ALT = alanine aminotransferase, AST = aspartate aminotransferase, BUN = blood urea nitrogen, CI = confidence interval, Cre = creatine, HB = hemoglobin, HE = hepatic encephalopathy, HR = hazard ratio, INR = international normalized ratio, PLT = platelet, PT = prothrombin, PTA = prothrombin activity, TBil = total bilirubin, TP = total protein, UGBI = upper gastrointestinal hemorrhage, WBC = white blood cell. After univariate Cox regression, clinically significant parameters were verified by multivariate analysis.

FER score (Table 2) = 0.01*HB-0.001*alpha-fetoprotein + 0.001*TBIL-0.019*PTA + 0.026*Age + 0.062*blood urea nitrogen (BUN) + 0.061*WBC + 0.996*infection (noted as 1, not noted as 0) + 1.127*HE (noted as 1, not noted as 0).

FRER score (Table 3) = 0.001*aspartate aminotransferase + 0.019*PT + 0.002*TBIL-0.029*PTA + 0.026*Age + 0.078*BUN + 0.059*WBC + 1.123*infection (noted as 1, not noted as 0) + 0.839*HE (noted as 1, not noted as 0).

MDCR (Table 4) score = 0.125*PT-0.001*alpha-fetoprotein + 0.007*TBIL-0.15*PTA + 0.022*Age + 0.073*BUN + 0.075*WBC + 1.096*Infection (noted as 1, not noted as 0) + 1.194*HE (noted as 1, not noted as 0).

### 3.3. Performance of the 3 new models

**Figure 3.**
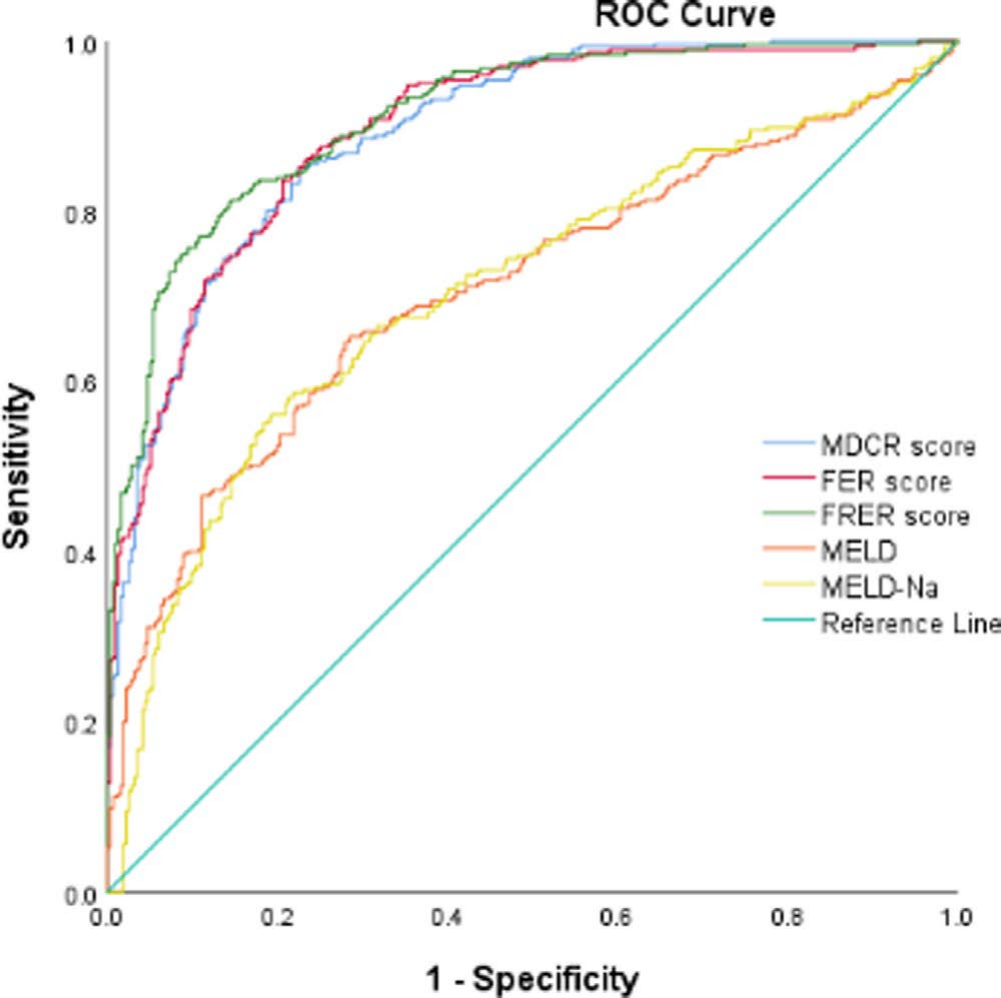
ROC curve of the HBV-ACLF prognostic model. The results showed that the model established by the indicators of the FRER (first reexamination results) had the largest area under the curve. HBV-ACLF = hepatitis B virus-related acute-on-chronic liver failure, ROC = receiver operating characteristic.

First, the performance of the FRER, FER, and MDCR scores was estimated (Fig. 3), and their areas under the ROC curve were 0.911, 0.895, and 0.893, respectively (Table 5). In addition, we compared the 3 new models and other formulas (including the MELD and MELD-Na scores) in predicting short-term prognosis. The MELD score was calculated using the following formula:^[12]^ 3.78*log_10_ [bilirubin (mg/dL)] + 11.2*log_10_ (INR) + 9.57*log_10_ [Scr (mg/dL)] + 6.43; the MELD-Na score was calculated based on the MELD score using the formula:^[13]^MELD- Na − (0.025 × MELD × (140 − Na)) + 140. The results showed that the model established by the indicators of the FRER had a larger area under the curve (AUROC = 0.911, 95% confidence interval [CI]: 0.889, 0.933) (Table 5).

**Table 5:** Area under the curve for each scoring system.

	AUROC	95% CI	*P*	Cutoff value	Sensitivity	Specificity
FRER score	0.911	(0.889, 0.933)	<.001	3.65	81.3%	85.5%
MDCR score	0.893	(0.868, 0.917)	<.001	2.85	77.7%	85.2%
FER score	0.895	(0.871, 0.919)	<.001	3.70	83.6%	79.6%
MELD	0.708	(0.667, 0.750)	<.001	24.38	64.6%	72.1%
MELD-Na	0.707	(0.666, 0.748)	<.001	27.13	58.7%	78.1%

Firstly, the performance of these 3 new scoring systems were estimated, and the largest of these areas under the ROC curve was 0.911. In addition, we compared the efficiency of the FRER score and other formulas (including FER, MDCR, MELD, MELD-Na scores) in predicting short-term prognosis. The results illustrated that the FRER score was superior to those models mentioned above.

AUROC = the area under the receiver operating characteristic, FER = first examination results, FRER = first reexamination results, MELD = model of end-stage liver disease, MDCR = mean daily change in these results.

**Figure 4.**
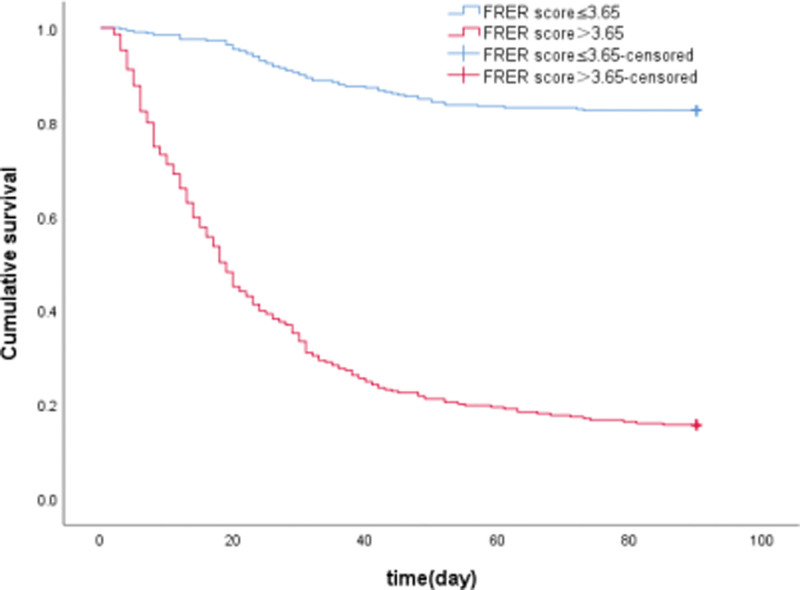
FRER score > or ≤ 3.65-censored means no endpoint events such as death or liver transplantation at the end of 90-day follow-up. The newly identified FRER score demonstrated its applicability in predicting a poor prognosis within 90 days. FRER = first reexamination results.

The newly founded FRER score showed its applicability in predicting a poor prognosis within 90 days. A cutoff point of the FRER score 3.65 was suggested to indicate a poor outcome, and it had an 81.3% sensitivity and 85.5% specificity. The results demonstrated that patients with a higher FRER score (>3.65) had an increased risk for poor outcome (Fig. 4). The transplant-free survival rate at 90 days were 15.7% (46/293) versus 82.4% (272/330) (*P* < .001) in groups of patients with FRER score >3.65 and ≤3.65, respectively.

## 4. Discussion

According to statistics, there are approximately 70 million cases of chronic hepatitis B carriers in China, including approximately 20 to 30 million cases of active hepatitis B.^[14]^ In China, HBV-ACLF accounts for nearly 80% of ACLF cases, and the high incidence of HBV infection can cause severe liver function damage as well as multiorgan failure.^[15]^ Although antiviral therapy is an effective treatment strategy that can improve the prognosis of patients with HBV-ACLF,^[16]^ its mortality rate is still high, and the 90-day mortality rate of HBV-ACLF in this study was 48.96%. ACLF is a dynamically progressive disease, and its clinical course can change within a few days. Moreover, the patient condition can gradually worsen or gradually improve, and the direction of disease change is closely related to the prognosis. However, studies on the dynamic changes in this disease are lacking. In this study, the FER and FRER were recorded and the MDCR was calculated. A multivariate Cox regression analysis of the FER, general conditions and complications was performed to screen out independent risk factors related to the patient short-term prognosis. The final results show that the FRER combined with the other FER and other corresponding indicators can better reflect the patient 90-day prognosis. The reason may be that after a short period of intervention, the patient condition changes and the change trend at this time is more closely related to the prognosis.

Total serum bilirubin (TBIL) reflects the metabolic function of the liver. Bilirubin is a metabolic product of heme, and there are 4 types of bilirubin in human blood: unconjugated bilirubin (UB), bilirubin monoglucuronide, bilirubin diglucuronide and δ bilirubin. UB is directly produced by the catabolism of heme, which is insoluble in water and does not covalently bind to albumin, and it is transported in the blood by attachment to albumin. Catalyzed by uridine diphosphate-glucuronosyltransferase in hepatocytes, UB binds glucuronosyl groups with covalent bonds to form bilirubin monoglucuronide and bilirubin diglucuronide, which are collectively referred to as conjugated bilirubin and can be soluble in water.^[17]^ When liver function is impaired,^[18]^ bilirubin metabolism is impaired and TBIL is elevated. The value of TBIL can be used to determine the severity of the disease, and its change value can assist in determining the direction of disease progression.

PT and PTA both reflect coagulation function. PT is an important indicator of the exogenous coagulation pathway, and its length reflects the plasma levels of PT and coagulation factors I, II, V, VII, and X. Moreover, PT is a more sensitive and commonly used indicator of the exogenous coagulation system.^[19]^ The liver is an important organ for the synthesis of coagulation factors, and when liver function is impaired, then a decrease in the synthesis or activity of coagulation factors will occur, which in turn will be accompanied by abnormalities in coagulation mechanisms and fibrinolysis functions and manifest as a variety of coagulation dysfunctions. PT will then be prolonged accordingly. PTA is a commonly used indicator of coagulation function in clinical practice. When the synthesis of coagulation factors decreases, PTA decreases significantly, and this characteristic is of great value for evaluating the condition and prognosis of ACLF patients.^[20]^ When hepatocytes are severely damaged, especially when they are necrotic, normal functioning hepatocytes are drastically reduced and the synthesis of coagulation factors drops drastically; thus, PTA decreases. Therefore, the level of PTA can reflect the degree of hepatocyte damage.

Several previous studies have shown that age is an independent risk factor for the prognosis of patients with ACLF, with greater age corresponding to worse prognosis.^[21–23]^ With age, the liver undergoes various changes, including a significant decrease in both liver volume and blood flow. In general, liver volume decreases by 20% to 40% in older adults,^[24]^ and it has been estimated that liver blood flow decreases by 35% to 50% in older adults, which may be associated with the age-related decrease in liver volume.^[25]^ The regenerative capacity of the liver also decreases with age.^[26,27]^ In addition, the immune response of the elderly to pathogens is low, and complications of infection are prone to occur during the course of the disease. The reserve function of various organs in elderly patients decreases significantly, which reduces tolerance to liver disease treatment.^[28]^

Common complications of ACLF include HE, hepatorenal syndrome, infection, ascites, and upper gastrointestinal hemorrhage. In this study, the prognosis of patients with combined HE and infection was significantly worse, with hazard ratios of 3.087 (2.384, 3.996) and 2.707 (1.414, 5.192), respectively, suggesting that infection and HE are both strong predictors of 90 days mortality in HBV-ACLF patients. Studies have shown that the systemic inflammatory response is a characteristic manifestation of ACLF. The white blood cell (WBC) count, plasma C-reactive protein levels and cytokine levels, such as interleukin (IL)-6, IL-1β, and IL-8, are all higher in patients with ACLF than in non-ACLF patients.^[29]^ When infection occurs, the levels of inflammatory cells, inflammatory factors and cytokines are further increased, leading to more severe liver damage and a worse prognosis.

There are some limitations in the present study that warrant consideration when interpreting our findings. Firstly, in this study only acute-on-chronic hepatitis B liver failure was studied, and liver failure caused by other types of hepatitis virus infection, drugs, and alcohol was not studied. Secondly, this is a retrospective study based on clinical data from single center, which may result in an increased possibility of selection bias and limit the generalization. Further international multi-center studies with more diversified patients should be conducted to substantiate our findings. Lastly, this study did not further validate the reliability of this prognostic model. Future work will be conducted in a multi-center prospective study to validate this prognostic model.

In conclusion, the prognostic evaluation model established using the FRER has the largest area under the ROC curve and the best predictive ability. This finding is different from several previous studies in which the FER were used to build prognostic prediction models for disease prognosis. This study innovatively used the FRER for analysis to provide insights and contributions to related fields.

## Author contributions

**Conceptualization:** Dakai Gan.

**Data curation:** Xiaoqing Zhang, Zhen Zhang, Longchuan Zhu.

**Funding acquisition:** Dakai Gan.

**Investigation:** Tao Long, Nengwen Xie.

**Methodology:** Yunfeng Xiong, Guoliang Feng.

**Resources:** Daya Luo.

**Software:** Bo Zou, Xuezhen Zhang.

**Validation:** Kaige Zhang, Yang He, Jiao Wan.

**Writing – original draft:** Yuyu Zeng.

**Writing – review & editing:** Molong Xiong.
